# Role of the elastography strain ratio using transrectal ultrasonography in the diagnosis of prostate cancer and clinically significant prostate cancer

**DOI:** 10.1038/s41598-022-25748-4

**Published:** 2022-12-07

**Authors:** Jeong Woo Yoo, Kyo Chul Koo, Byung Ha Chung, Kwang Suk Lee

**Affiliations:** Department of Urology, Gangnam Severance Hospital, Yonsei University College of Medicine, 211 Eonju-Ro, Gangnam-Gu, Seoul, 06273 Republic of Korea

**Keywords:** Prostate cancer, Prostate, Cancer imaging

## Abstract

This study investigated the efficacy of the elastography strain ratio (ESR) as a predictor of prostate cancer (PCa) in targeted prostate biopsy. In total, 257 patients who underwent magnetic resonance imaging-targeted biopsy were enrolled. Before biopsy, we placed regions of interest (zone A and B) in the lesion and levator ani. The ESR was measured as zone A/zone B. Multivariate analyses were performed to predict PCa and clinically significant PCa. There were 206 (71.5%) positive cancer lesions. No difference in digit rectal examination findings was found between patients with and without PCa. For predicting clinically significant PCa, an ESR ≥ 6.8 was significantly higher in the PCa (+) group than in the PCa (−) group (*p* < 0.001). The area under the receiver operating characteristic curve (AUC) for the conventional variables (model 1) plus the ESR was 0.845, which was significantly higher than that for model 1 (*p* = 0.001). In prostate imaging reporting and data system score 3 lesions, an ESR ≥ 4.6 was a significant predictor of PCa (*p* = 0.002). The AUC in model 1 plus the ESR was 0.856, which was significantly higher than that in model 1 alone (*p* = 0.017). The ESR is useful for predicting clinically significant PCa.

## Introduction

Prostate-specific antigen (PSA) and digital rectal examination (DRE) are standard tools for assessing the risk of prostate cancer^1^. Overcoming the low diagnostic rate of conventional biopsy and increasing the clinically significant prostate cancer diagnosis rate, recent guidelines recommend multiparametric magnetic resonance imaging (MRI) or new biomarkers (such as the Prostate Health Index, SelectMDx, 4Kscore, and ExoDx Prostate test) to precisely select the patients who should undergo prostate biopsy.

DRE is a conventional method for detecting prostate cancer early; however, it is a subjective test and has low representativeness and reproducibility^2^. During DRE, it is difficult to palpate anterior cancer, and this examination has low diagnostic performance, especially sensitivity^3,4^. Therefore, DRE is not useful for the diagnosis of anterior site prostate cancer. The cancer detection rate is increasing through pre-biopsy prostate MRI, which has recently been highlighted at an anterior site that was not initially performed in the conventional 12 cores biopsy^1,5^. In contrast, several embedded functions of transrectal ultrasonography (TRUS), which are technologically advanced and widespread, have the potential to assist DRE. One of the embedded functions of ultrasonography is elastography, which measures the tissue stiffness^4,6,7^. This technology presents different colors and numerical data according to tissue stiffness. The strain ratio is calculated as the ratio of the numerical data used to measure the stiffness of the different tissues.

Elastography has proven to be useful for the diagnosis of prostate cancer^8^. Conventional elastography is a qualitative test that has not yet been widely used. However, it can be quantified using the elastography strain ratio (ESR)^4^. Hard palpation in DRE suggests cancer; however, previous studies on the diagnosis of prostate cancer using the ESR are limited. Therefore, this study aimed to investigate the efficacy of the ESR as a predictor of prostate cancer and clinically significant prostate cancer in MRI-targeted prostate biopsy.

## Results

### Baseline characteristics of the patients who underwent MRI-targeted prostate biopsy

Of 257 patients, 207 (80.5%) were diagnosed with prostate cancer (Table 1). Of the patients with prostate cancer, 15 (7.2%) were diagnosed based on target biopsy results with concomitant negative systemic biopsy findings. Patients in the prostate cancer (+) group were significantly older, had a higher PSA level, and had a higher proportion of positive family history than those in the prostate cancer (−) group. No difference in DRE findings was found between patients with and without prostate cancer. Of 288 target lesions, 206 (71.5%) and 174 (60.4%) were diagnosed as prostate cancer and clinically significant prostate cancer, respectively (Table 2). The ESR in the overall target lesions showed no significant difference between the prostate cancer (−) and prostate cancer (+) groups (3.38 and 3.23, respectively, *p* = 0.100). The median ESR of clinically significant prostate cancer (+) group and significant prostate cancer (−) group were 3.41 (2.11–4.72) and 3.28 (2.20–4.99) respectively, and it was not statistically significant difference (*p* = 0.092). The median ESRs of clinically significant prostate cancer diagnosed at the anterior and posterior sites were 3.61 (2.56–5.38, n = 69) and 3.10 (2.05–4.90, n = 105), respectively; these were not significantly different (*p* = 0.193).

**Table 1:** Baseline characteristics of patients who underwent prostate MRI-targeted biopsy.

	Prostate cancer (−)	Prostate cancer (+)	*p*-value
No. of patients	50 (19.5)	207 (80.5)	
Age (y)	67.1 ± 7.7	70.1 ± 8.0	0.017
PSA level (ng/mL)	6.05 (4.85–9.90)	8.15 (5.30–14.54)	0.002
Prostate volume (cm^3^)	42.6 (32.2–53.5)	30.2 (23.2–41.4)	< 0.001
PSA density (ng/mL/cm^3^)	0.16 (0.10–0.21)	0.27 (0.17–0.53)	0.000
Prostate biopsy history	15 (30.0)	20 (9.7)	0.004
Family history	0 (0.00	9 (4.3)	0.004
DRE (+)^a^	13 (26.0)	57 (27.5)	0.875
**Positive systemic biopsy finding**		192 (92.8)	
Gleason grade group			
1		35 (16.9)	
2–3		79 (38.2)	
4		69 (33.3)	
5		9 (4.3)	

Data are presented as number (%), mean ± standard deviation, and median (interquartile range).

^a^DRE ( +) is defined as a palpable nodule or hard surface.

DRE, digital rectal exam; PSA, prostate-specific antigen.

**Table 2:** Baseline characteristics of target lesions that underwent prostate MRI-targeted biopsy.

	Prostate cancer (−)	Prostate cancer (+)	*p*-value
No. of target lesions	82 (28.5)	206 (71.5)	
**Site (posterior/anterior)**			0.145
Posterior	53 (64.6)	114 (55.3)	
Anterior	29 (35.4)	92 (44.7)	
**Site (base/mid-gland/apex)**			0.508
Base	12 (14.6)	28 (13.6)	
Mid-gland	45 (54.9)	123 (59.7)	
Apex	25 (30.5)	55 (26.7)	
Size (cm)	0.8 (0.6–1.3)	1.3 (0.9–2.0)	< 0.001
**PI-RADS score**			< 0.001
3	37 (63.8)	21 (36.2)	
4	42 (30.2)	97 (69.8)	
5	3 (3.3)	88 (96.7)	
**Gleason grade group**			
1		32 (15.5)	
2–3		101 (49.0)	
4		60 (29.1)	
5		13 (6.3)	
**Elastography strain ratio**	3.38 (2.09–4.64)	3.23 (2.18–4.95)	0.100
≥ 6.3^a^	3 (3.7)	24 (11.7)	0.010
≥ 6.8^b^	0 (0.0)	21 (10.2)	< 0.001

Data are presented as number (%), mean ± standard deviation, and median (interquartile range).

^a^ Cut-off value of the elastography strain ratio for predicting prostate cancer using the Youden index.

^b^ Cut-off value of the elastography strain ratio for predicting clinically significant prostate cancer using the Youden index.

MRI, magnetic resonance imaging; PI-RADS, Prostate Imaging Reporting and Data System.

Interestingly, for predicting clinically significant prostate cancer, an ESR ≥ 6.8 was significantly higher in the prostate cancer (+) group than in the prostate cancer (−) group (0.0% vs. 10.2%, *p* < 0.001). There was no statistically significant difference in ESR between the DRE (+) group and the DRE (−) group using the Mann–Whitney U test (*P* = 0.295).

### ESR as a risk factor for predicting clinically significant prostate cancer

Results of univariable analysis showed that age, the PSA level, prostate volume, prostate biopsy history, Prostate Imaging Reporting and Data System (PI-RADS) score, and an ESR ≥ 6.8 were potential factors for predicting clinically significant prostate cancer. An abnormal DRE finding was not a predictive factor in this study. In the multivariate analysis of model 2 using an ESR ≥ 6.8 in addition to conventional variables (model 1: age, PSA level, prostate volume, prostate biopsy history, and PI-RADS score), an ESR ≥ 6.8 was identified as showing an increased OR for predicting clinically significant prostate cancer (Table 3). The actual rate of clinically significant prostate cancer in ESR ≥ 6.8 was 87.0%.

**Table 3:** Results of multivariable logistic regression analyses for predicting clinically significant prostate cancer on targeted biopsy.

Clinically significant prostate cancer	Model 1		Model 2	
	Odds ratio (95% CI)	*p*-value	Odds ratio (95% CI)	*p*-value
Age	1.04 (0.970–1.064)	0.132	1.12 (0.996–1.263)	0.257
PSA level	1.06 (1.010–1.123)	0.014	1.14 (1.023–1.127)	0.018
Prostate volume	0.90 (0.921–0.959)	< 0.001	0.89 (0.847–0.956)	0.003
Prostate biopsy history	0.74 (0.338–1.721)	0.488	0.72 (0.321–1.786)	0.572
**PI-RADS score**				
3	4.78 (2.692–6.233)	< 0.001	4.38 (1.872–8.896)	< 0.001
4	6.45 (3.868–11.426)	< 0.001	6.23 (3.423–9.986)	< 0.001
5	13.37 (5.326–27.233)	< 0.001	12.13 (4.974–34.472)	< 0.001
Elastography strain ratio ≥ 6.8^a^			15.14 (2.859–126.339)	0.031

Data are presented as median (interquartile range).

^a^ Cut-off value of the strain ratio predicting clinically significant prostate cancer using the Youden index.

CI, confidence interval; PI-RADS, Prostate Imaging Reporting and Data System; PSA, prostate-specific antigen.

**Figure 1:**
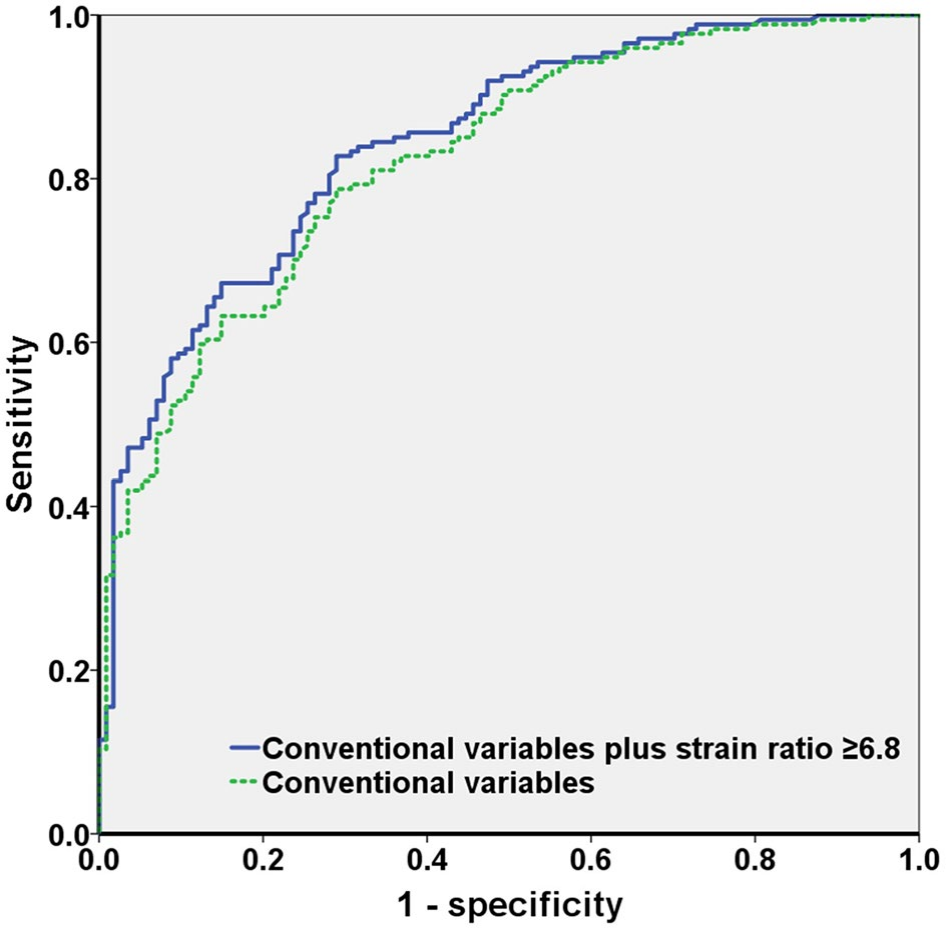
ROC curves of detecting clinically significant prostate cancer lesion using clinical variables and the elastography strain ratio. ROC, receiver operator characteristic.

The area under the receiver operating characteristic curve (AUC) in model 2 was 0.845 (95% CI: 0.800–0.889), which was significantly higher than that in model 1 (0.824, 95% CI: 0.776–0.871) (*p* = 0.001) (Fig. 1). The sensitivity, specificity, positive predictive value, and negative predictive value of an ESR ≥ 6.8 were 12.0%, 97.3%, 81.0%, and 53.2%, respectively.

### Efficacy of ESR for stratifying lesions with PI-RADS score 3

In PI-RADS score 3 lesions, results of univariable analysis for prostate cancer showed that the PSA level, prostate volume, and an ESR ≥ 4.6 were potential predictive factors of prostate cancer. In the multivariate analysis of model 2, an ESR ≥ 4.6 in addition to conventional variables (model 1: PSA level and prostate volume), an ESR ≥ 4.6 remained a significant predictor of prostate cancer (OR = 5.71, *p* = 0.002) (Table 4). Among PI-RADS score 3 lesions, the actual rate of prostate cancer in ESR ≥ 4.6 was 75.0%.

**Table 4:** Results of multivariable logistic regression analyses for predicting prostate cancer on prostate MRI-targeted biopsy of PI-RADS score 3 lesions.

Cancer (PI-RADS score 3)	Model 1		Model 2	
	Odds ratio (95% CI)	*p*-value	Odds ratio (95% CI)	*p*-value
PSA	1.21 (1.013–1.441)	0.035	1.26 (1.035–1.539)	0.021
Prostate volume	0.92 (0.865–0.971)	0.003	0.91 (0.856–0.967)	0.002
Elastography strain ratio ≥ 4.6^a^			5.71 (1.285–25.369)	0.002

Data are presented as median (interquartile range).

^a^Cut-off value of the elastography strain ratio for predicting prostate cancer using the Youden index.

CI, confidence interval; MRI, magnetic resonance imaging; PI-RADS, Prostate Imaging Reporting and Data System; PSA, prostate-specific antigen.

**Figure 2:**
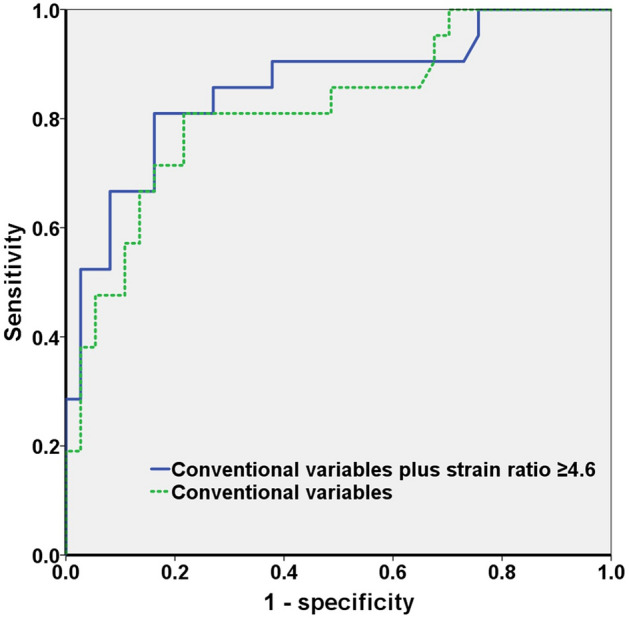
ROC curves of detecting prostate cancer lesion using clinical variables and the elastography strain ratio in PI-RADS score 3 lesions. PI-RADS, Prostate Imaging Reporting and Data System; ROC, receiver operator characteristic.

The AUC in model 2 was 0.856 (95% CI: 0.749–0.964), which was significantly higher than that in model 1 (0.818, 95% CI: 0.701–0.935) (*p* = 0.017) (Fig. 2). The sensitivity, specificity, positive predictive value, and negative predictive value of an ESR ≥ 4.6 were 53.3%, 74.4%, 42.1%, and 82.1%, respectively.

## Discussion

The ESR is a predictor of clinically significant prostate cancer in target lesions, and an ESR ≥ 6.8 was presented as the cut-off value. Additionally, an ESR ≥ 4.6 in PI-RADS score 3 lesions on prostate MRI was presented as a predictor of prostate cancer.

Malignant tumors are stiffer than normal tissue. Benign prostatic hyperplasia or normal prostate has a glandular cavity and a homogeneous internal texture, but cancer cells exhibit a stroma reaction in which the normal glandular tissue is destroyed by cancer cell invasion^4^. In cancer tissues, the density of cancer cells is increased^9^. Consequently, malignant tumors are stiffer than benign lesions; therefore, DRE is recognized as a screening test for prostate cancer. However, palpation may not be possible depending on the location of the tumor, is highly subjective, and may not be reproducible or representative.

Our study is valuable and scalable, as it is one of the few studies that applied the ESR, which is widely used in other cancers, to the prostate cancer diagnosis field. In breast, thyroid, and pancreatic cancers, elastography is recognized as a promising technique because cancer tissue is stiffer than normal tissue^10^. Breast cancer has been actively studied for a long time^11^, and recently, elastography has also been studied in the differentiation of benign and malignant soft tissue tumors^12^, predicting malignant thyroid nodules^13^, detecting pancreatic cancer using endoscopic ultrasonography^14,15^, and assessing liver disease^16^.

Several studies have demonstrated the diagnostic performance of elastography for prostate cancer. In 2018, Tyloch et al. reported a review of six meta-analyses that evaluated the use of elastography in the diagnosis of prostate cancer^17^. According to this study, the meta-analysis by Sang et al. showed that the highest diagnostic performance, sensitivity, and specificity were 0.844 (range, 0.696–0.927) and 0.860 (range, 0.792–0.908)^2^. The diagnostic performance of this previous review is superior to that of our study, especially in terms of sensitivity. However, previous reviews did not stratify the methods between shear wave elastography and real-time elastography. In contrast to our study, which made comparisons per target lesion, previous studies have confirmed the diagnostic performance of elastography with histopathological findings from TRUS random biopsy or radical prostatectomy specimens. To our knowledge, no previous study has confirmed the pathologic result of MRI target lesions and analyzed it using logistic regression. In our study, as a result of logistic regression analyses of an ESR ≥ 6.8 and the conventional variables, we found that an ESR ≥ 6.8 indicated an increased risk for clinically significant prostate cancer.

The ESR is helpful in the differential diagnosis of prostate cancer in PI-RADS score 3 lesions on prostate MRI. Multiparametric MRI is a powerful modality for detecting clinically significant prostate cancer^18^, and current guidelines recommend pre-biopsy prostate MRI^1^. This improves the diagnostic performance for clinically significant prostate cancer and reduces the rate of unhelpful prostate biopsies. However, the diagnostic accuracy of PI-RADS score 3 lesions is poor^19^. Our results showed that an ESR ≥ 4.6 is a predictor of prostate cancer. In clinically significant prostate cancer, the cut-off value of the ESR was ≥ 5.6, which was statistically significant in univariable logistic regression analysis; however, it was not statistically significant in multivariable logistic repression analyses.

Recently, besides ultrasound elastography, MRI elastography has been studied^20^. Prostate MRI is a powerful diagnostic evaluation tool, such as PI-RADS, whereas MRI elastography plays a supporting role and is not useful as a screening test. A previous study reported that pre-biopsy prostate MRI is only used in 22% of biopsy-naive patients because of its high cost^21^. Alternatively, the CADMUS trial studied the diagnostic performance of multiparametric ultrasonography. The objective of this trial is agreement in recognization of lesion between multiparametric ultrasound and multiparametric MRI. Multiparametric ultrasound consisted of conventional B-mode images, high frequency or fine flow Doppler images, real-time elastography, and contrast enhanced images^21^. Yet, these data were also qualitative, such as the PI-RADS. In our previous study, we analyzed grayscale as a quantitation of hypoechoic lesions among qualitative variables (hypoechoicity, irregularity, microcalcification, and vascularity), and reported that quantitative scoring is useful for detecting prostate cancer^22^. This study allowed us to quantify tissue stiffness.

The lack of previous studies on the ESR in prostate cancer diagnosis is presumably due to the difficulty in selecting a tissue to serve as a reference point. We also considered the urinary bladder, normal prostate tissue, and pelvic floor muscle as reference points. There was concern that the stiffness of the bladder would be measured differently depending on the amount of urine inside the bladder, and prostate tissue thought to be normal was excluded because of the possibility of hidden cancer. Among the pelvic floor muscles, the target lesion and levator ani close to the prostate were set as reference points for easy measurement on one screen.

Our study is valuable in suggesting the cut-off value of the ESR and its diagnostic performance and usefulness. We expect better diagnostic performance when combining the ESR and grayscale of hypoechoic lesions using ultrasonography. However, there are also limitations to pilot studies such as ours. First, depending on the ESR mechanism, different results may be obtained depending on the equipment and physician. We are currently preparing a prospective study to validate our results but have not yet conducted an internal validation. If a large cohort study is conducted in the future, the cut-off value may be different, but a similar trend is expected. Second, we used the levator ani as a reference point for comparison with cancer tissue, but many studies are needed to determine a unified reference point. Third, since our study analyzed specimens from MRI-targeted prostate biopsy, it cannot be free of patient selection bias or operator bias. It is necessary to confirm whether there is a difference with the surgical specimen. In addition, it is necessary to set the target lesion during the screening stage before performing MRI, to collect and analyze data. In patients who have not undergone pre-biopsy prostate MRI, it is difficult to reproduce our method when no target lesions are identified in TRUS. Fourth, tissue stiffness may differ depending on the tumor location and aggressiveness. Owing to the small number of target lesions in our study, further analysis was difficult, and tissue stiffness will need to be verified in future studies. Finally, for a high positive predictive value, we set a cut-off value of high specificity as a predictor of prostate cancer and thus showed low sensitivity. Large data sets and well-controlled prospective multicenter studies are needed to confirm our findings including usefulness of ESR in PI-RADS score 3 lesions and address this issue.

In conclusion, the PSA level and DRE are still recommended as screening tests for prostate cancer and recent guidelines recommend pre-biopsy prostate MRI in patients with suspected prostate cancer. However, MRI is unsuitable for screening due to its high cost and the need for equipment. We evaluated the ESR of the target lesion in MRI-targeted prostate biopsy and confirmed its potential as a complementary diagnostic tool. This retrospective pilot study had some limitations, and additional prospective studies in pre-biopsy prostate MRI setting are needed to evaluate the potential role of the ESR. Currently, it is difficult to apply in a clinical setting without pre-biopsy prostate MRI, but TRUS using elastography combined with the grayscale of hypoechoic lesions is expected to be a better screening test for prostate cancer.

## Methods

### Ethical approval

This study was approved by the Institutional Ethics Committee (Yonsei University Health System, Seoul, Korea; approval number: 3-2021-0313), and all procedures were conducted in accordance with the ethical standards of the 1964 Declaration of Helsinki and its later amendments. The requirement for informed consent was waived by the ethics committee of Yonsei University Health System because this study was based on retrospective, anonymous patient data and did not involve patient intervention or the use of human tissue samples.

### Patient selection and data collection

This study reviewed the data of 264 patients who underwent MRI-targeted prostate biopsy at our institution between January 2020 and September 2021. Seven patients were excluded from the study for the following reasons: (i) four patients had a diagnosis of prostate cancer with active surveillance, (ii) two patients had a history of radiotherapy, and (iii) one patient had a diagnosis of transitional cell carcinoma. The remaining 257 patients were included in the study.

Patient characteristics were obtained, including clinicopathological data such as age, serum PSA level, prostate volume, prostate biopsy history, prostate cancer family history, DRE findings, MRI findings, number of positive biopsy cores, and pathologic outcomes. Family history of prostate cancer was defined as having a father and/or one or more brothers with a diagnosis of prostate cancer. Men whose family history could not be determined, such as those with no brothers or whose fathers died early, were considered to have a negative family history. Clinically significant prostate cancer was defined as a Gleason grade ≥ 2. All MRI findings were evaluated by an experienced urologic-radiologist and graded according to PI-RADS version 2.1^23^. The presence of visible lesions was defined as lesions with a PI-RADS score ≥ 3. The target lesion site was divided into anterior and posterior lesions, based on the location of the urethra.

### Magnetic resonance imaging-targeted prostate biopsy procedure

All prostate biopsies were performed after the periprostatic nerve block procedure using a Chiba needle (A & A M.D. Inc., Seongnam, Korea). First, four MRI-targeted core biopsies for each targeted lesion were performed, followed by 12-core systemic biopsies. The prostate biopsy was performed using a BK3000 ultrasound system (BK Medical, Peabody, MA, USA) with a 7.5–12 MHz multiplanar probe, and the MRI-targeted prostate biopsy was performed using an MRI/TRUS image-based fusion program (BioJet; GeoScan, Lakewood Ranch, FL, USA). Since the fusion program marked all target lesions, it was possible to measure ESR for target lesions that are difficult to identify in TRUS.

All biopsies were performed using guide channels at 19° to the transducer axis of the side-fire probe (BK Medical, Peabody, MA, USA) and an 18-gauge, 20-cm disposable core biopsy instrument (Max-Core®, CR Bard Inc., New Providence, NJ, USA).

### ESR measurement

Elastography strain images are created by applying pressure to the tissue using a probe. At the same pressure, stiff and soft regions are presented by the color code map. This is an additional extension to the existing B-mode image. This technique requires some learning curve. It adds only a few minutes of extra time to the procedure. This function must be embedded into the ultrasound system. Depending on the equipment, shear wave elastography or strain elastography may be supported, or both may or may not be supported. As with shear wave elastography, quantitative elasticity measurements cannot be obtained; therefore, the relative stiffness difference with the reference tissue is presented as a "ratio”^4,24^.

**Figure 3:**
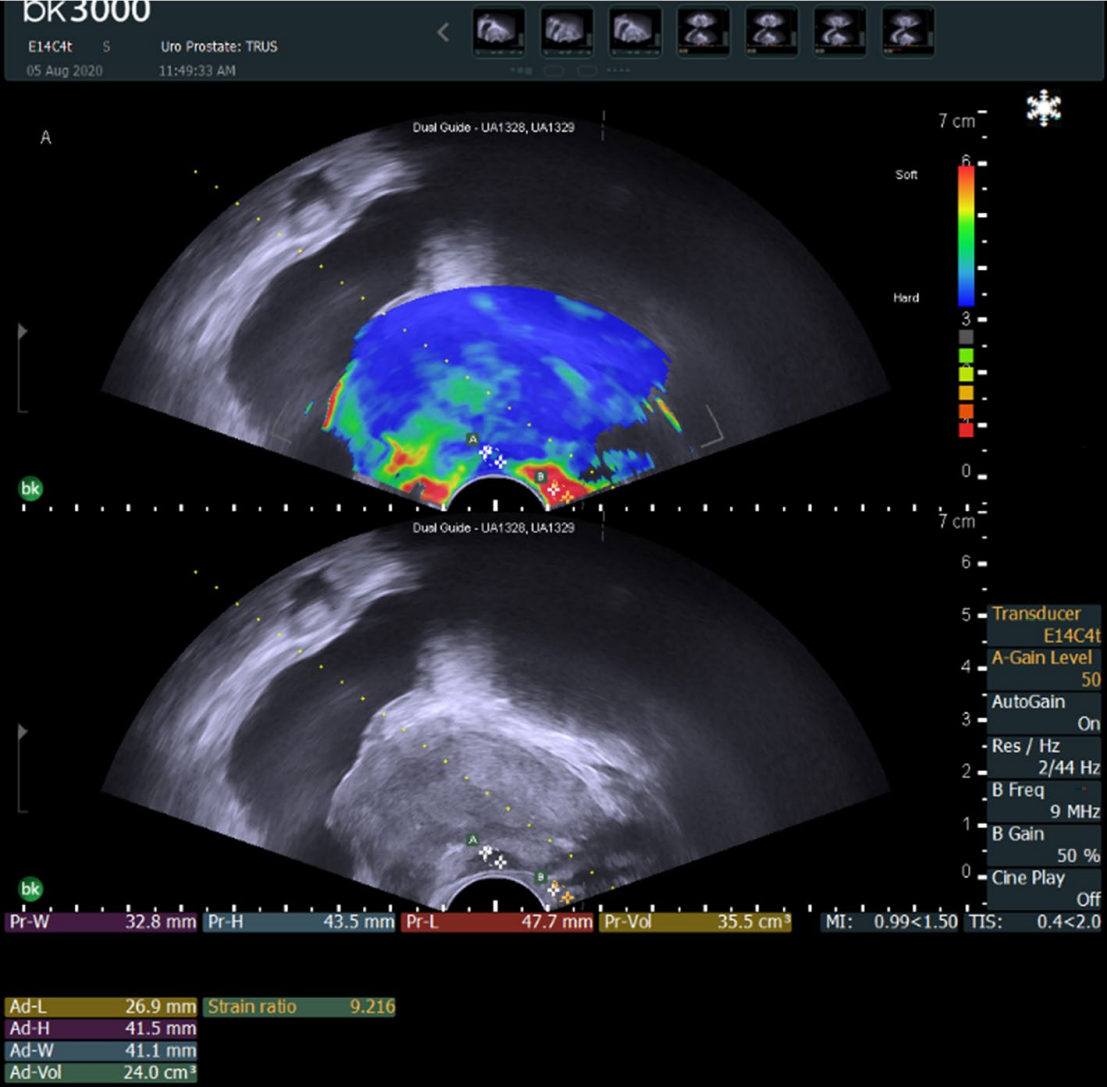
Elastography strain ratio (zone A/zone B). (**A**) Target lesion, (**B**) levator ani.

Before the biopsy procedure, the physician measured the ESR using a function embedded in the ultrasound system. After confirming the target lesion with B-mode ultrasound, the physician set the elastography color-coded map to include a sufficient area of the whole prostate concomitant with real-time ultrasonography. The color-coded scale includes red and green for softer areas and blue for stiffer areas. To measure the ESR, we placed regions of interest (zone A and zone B, respectively) in the target lesion and levator ani. The ESR was measured as (zone A/zone B) (Fig. 3). All images of the target lesions in TRUS were stored in conjunction with matching MRI images and ESR ultrasonograms using a picture archiving and communication system (GE Healthcare, Barrington, IL, USA).

### Statistical analysis

Continuous variables are expressed as mean ± standard deviation or median (interquartile range). Categorical variables are reported as the number of occurrences and frequency. The Pearson χ^2^ test was used to statistically compare continuous and categorical variables. The Mann–Whitney U test was used to confirm correlation of ESR and positive DRE. ROC curves and AUCs were used to calculate the performance of the ESR as an independent predictor of prostate cancer. To specify the quantitation range of the ESR for target lesions, the cut-off value was assessed from the AUC. These optimal cut-off values were based on analyses using the Youden index (sensitivity + specificity–1). Univariate and multivariate logistic regression analyses were used to predict prostate cancer and clinically significant prostate cancer. To identify the increased diagnostic performance of the ESR for target lesions, we compared it to conventional variables, including age, the PSA level, prostate volume, abnormal DRE findings, prostate biopsy history, family history, and PI-RADS score. Pairwise comparisons of ROC curves were conducted.

All statistical comparisons were performed using SPSS version 26 (IBM Corporation, Armonk, NY, USA) and MedCalc version 11.6 (MedCalc Software, Ostende, Belgium). Statistical significance was set at a *p-*value < 0.05.

## Data Availability

The datasets used and analyzed during the current study are available from the corresponding author upon reasonable request.
